# Vitamin D ameliorates age-induced nonalcoholic fatty liver disease by increasing the mitochondrial contact site and cristae organizing system (MICOS) 60 level

**DOI:** 10.1038/s12276-023-01125-7

**Published:** 2024-01-04

**Authors:** Gyu Hee Kim, Hyeon-Ju Jeong, Yoo Jeong Lee, Hyeon Young Park, Soo Kyung Koo, Joo Hyun Lim

**Affiliations:** Division of Endocrine and Kidney Disease Research, Department of Chronic Disease Convergence Research, Korea National Institute of Health, Korea Disease Control and Prevention Agency, Cheongju, Chungbuk 28159 Republic of Korea

**Keywords:** Metabolic disorders, Mechanisms of disease

## Abstract

Nonalcoholic fatty liver disease (NAFLD) is the most common liver disease. Despite intensive research, considerable information on NAFLD development remains elusive. In this study, we examined the effects of vitamin D on age-induced NAFLD, especially in connection with mitochondrial abnormalities. We observed the prevention of NAFLD in 22-month-old C57BL/6 mice fed a vitamin D_3_-supplemented (20,000 IU/kg) diet compared with mice fed a control (1000 IU/kg) diet. We evaluated whether vitamin D_3_ supplementation enhanced mitochondrial functions. We found that the level of mitochondrial contact site and cristae organizing system (MICOS) 60 (Mic60) level was reduced in aged mice, and this reduction was specifically restored by vitamin D_3_. In addition, depletion of *Immt*, the human gene encoding the Mic60 protein, induced changes in gene expression patterns that led to fat accumulation in both HepG2 and primary hepatocytes, and these alterations were effectively prevented by vitamin D_3._ In addition, silencing of the vitamin D receptor (VDR) decreased the Mic60 levels, which were recovered by vitamin D treatment. To assess whether VDR directly regulates Mic60 levels, we performed chromatin immunoprecipitation and reporter gene analysis. We discovered that VDR directly binds to the *Immt* 5’ promoter region spanning positions −3157 to −2323 and thereby upregulates Mic60. Our study provides the first demonstration that a reduction in Mic60 levels due to aging may be one of the mechanisms underlying the development of aging-associated NAFLD. In addition, vitamin D_3_ could positively regulate Mic60 expression, and this may be one of the important mechanisms by which vitamin D could ameliorate age-induced NAFLD.

## Vitamin D: A New Hope in Combating Age-Induced Fatty Liver Disease

Nonalcoholic fatty liver disease (a condition in which fat builds up in the liver) (NAFLD) is increasingly affecting health, especially among the older population. The causes behind this disease are still unclear, but a fresh study by Lim and colleagues adds to our knowledge. They discovered that a reduction in a protein named Mic60 in the liver mitochondria of old mice leads to fat buildup, a major sign of NAFLD. However, when the mice consumed a diet enriched with vitamin D3, the Mic60 protein levels returned to normal, thus avoiding fat buildup. The study also showed that vitamin D3 directly controls Mic60 levels, implying that having enough vitamin D could help prevent NAFLD onset, particularly in elderly people.

This summary was initially drafted using artificial intelligence, then revised and fact-checked by the author.

## Introduction

Nonalcoholic fatty liver disease (NAFLD) is diagnosed when simple intrahepatic fat deposition (steatosis) exceeds 5% of the liver weight. When steatosis is combined with severe inflammation, the accumulation of ROS and fibrosis development trigger nonalcoholic steatohepatitis (NASH) and cirrhosis, which ultimately leads to hepatocellular carcinoma^1^. Therefore, hepatocellular lipid metabolism is tightly regulated and largely leads to two outcomes: lipid acquisition or elimination. When the uptake of FFAs via FFA transporters, such as CD36, or de novo lipogenesis (DNL) in hepatic cells exceeds the export of FFAs via very-low-density lipoprotein (VLDL) packaging or the usage of FFAs by mitochondrial β-oxidation, hepatocellular fat gradually accumulates, which leads to NAFLD. Many factors are involved in regulating and maintaining hepatic lipid homeostasis^2^; however, the mechanisms involved in lipid homeostasis and the causes of its disruption largely remain to be characterized. Physiological changes due to aging are usually accompanied by metabolic syndromes, such as obesity, dyslipidemia, and insulin resistance, which are closely associated with NAFLD^3^. The accumulation of visceral fat caused by aging increases the FFA levels in the blood and induces FFA absorption by the liver^4^. In addition, the age-dependent reduction in growth hormone/insulin-like growth factor-1 reactions leads to DNL^5^. In contrast, aging decreases the expression of carnitine palmitoyl transferase-1 (CTP-1), the key enzyme regulating mitochondrial β-oxidation^6^. TG-VLDL is complexed with apoprotein B (apoB) and exported into the blood under tight regulation by insulin^7^. An increase in insulin resistance with aging disrupts TG export. Together, these aging-related dysfunctions accelerate the accumulation of TG in the liver, which is a typical phenomenon of NAFLD.

As vitamin D prevents various diseases, such as immune diseases and diabetes, many studies on vitamin D have been performed in recent years. The major stable circulating form of vitamin D in blood is 25-hydroxyvitamin D_3_ (25(OH)D_3_; that is, vitamin D_3_). It is generated via cytochrome P450 family 2 subfamily R member 1 (Cyp2R1) in the liver by hydroxylation at the C-25 position of 7-dehydrocholesterol, a nonenzymic product, in the skin mediated by sunlight. The active form of vitamin D in vivo is 1,25(OH)_2_-VitD_3_ (1,25VitD_3_), which is produced in the kidney. 1,25VitD_3_ binds a nuclear receptor, vitamin D receptor (VDR), thereby regulating many physiological processes, including immune responses and calcium homeostasis. 1,25VitD_3_ is ultimately catabolized in the kidney by cytochrome P450 family 24 subfamily A member 1 (Cyp24A1)^8^. Because 1,25VitD_3_ shows hormonal activity, the cellular mechanism is crucial for maintaining an appropriate amount of 1,25VitD_3_ by balancing metabolic anabolism and catabolism. The production of 7-dehydrocholesterol declines with age, and the amount of vitamin D in the blood gradually decreases with age^9^. Therefore, most elderly individuals are vitamin D deficient, with the degree of deficiency differing by race and sex^10^. Although many studies have shown inverse correlations between serum vitamin D_3_ levels and NAFLD^11^, the therapeutic effects of vitamin D on NAFLD remain controversial^12^.

Mitochondria are very dynamic organelles. Mitochondrial fusion and fission occur continuously and are precisely regulated^13^. In these processes, most mitochondrial proteins are synthesized in the cytosol and transported into mitochondria through unique translocase complexes located in the outer or inner mitochondrial membranes: translocase of the outer mitochondrial membrane (TOM), sorting and assembly machinery (SAM) in the mitochondrial outer membrane (OM), and translocase of the inner mitochondrial membrane (TIM) in the mitochondrial inner membranes (IM)^14^. To prevent the generation of misfolded or incorrectly targeted proteins, the unique mitochondrial unfolded protein response (mtUPR) or mitochondrial quality control system (MQC) is evoked^15^. Mitochondria are key intracellular organelles that generate energy and are mainly responsible for the β-oxidation of FFAs and ROS production during oxidative phosphorylation (OXPHOS). Therefore, in recent years, multiple studies have found an association between mitochondrial dysfunction and many chronic diseases, including NAFLD^16^. For example, Takeochi Y et al. reported that the specific depletion of mitochondrial fission factor (MFF), a mitochondrial fission regulator in the liver, causes high-fat diet-induced NASH^17^. Although many studies have intensively investigated the link between mitochondrial dysfunction and chronic diseases, the molecular mechanisms have still not been clearly explained.

In contrast to the simple OM structure, the IM is composed of two distinct regions: the linear-shaped inner boundary membrane (IBM) and winding-shaped crista membrane (CM). At the entrance point of cristae, two membranes meet to create narrow bottleneck-like structures called cristae junctions (CJs)^18^. In 2011, the Neupert W. group was the first to report the discovery of a protein complex essential for the maintenance and formation of cristae, namely, the mitochondrial contact site and organizing system (MICOS)^19^. The MICOS complex comprises two subcomplexes, Mic60-Mic19-Mic25 and Mic10-Mic26-Mic27, with Mic13 (Qil1) being a stabilizer of the Mic60 and Mic10 subcomplexes in humans. TIM complexes are localized in the IBM. In contrast, MICOS complexes are located in CJs, and OXPHOS complexes are localized in the CM. Most notably, ATP synthase (complex V) is located at the CM tip. Moreover, depletion of Mic60 (also known as mitofusin, inner mitochondrial membrane protein (IMMT), and MINOS2), Mic10 (MINOS1) and Atp21, a subunit of ATP synthase, disrupts normal crista structures and sequentially disturbs mitochondrial function^20^. Although Mic10 forms the structural core of MICOS, Mic60 is the main link between OM and IM, which are connected via TOM, voltage-dependent anion channels (VDACs) and SAM. In particular, Mic60 interacts with Sam50, a SAM component, to form the MICOS-SAM supercomplex, which is also called the mitochondrial intermembrane space-bridging complex (MIB)^21^. In addition, Mic60 is associated with PTEN-induced kinase 1 (PINK1), a key protein involved in mitophagy^22^. Additionally, several proteins that regulate mitochondrial dynamics interact with Mic60. For instance, mitochondrial dynamin-like GTPase optic atrophy 1 (Opa1) or SLC25A46, which is involved in mitochondrial fusion, interacts with Mic60, and these interactions are believed to be involved in the maintenance and formation of cristae^23,24^. Additionally, via its interaction with Mic60, Opa1 may tighten CJs and thereby prevent the release of cytochrome c, which is usually located within cristae. Oma1, a stress-inducible peptidase and a major regulator of mitochondrial fission, is thought to promote apoptosis^25^. In contrast, Viana MP et al. recently reported that Oma1 stabilizes OM–IM supercomplexes by interacting with Mic60 in an Opa1-independent manner and that the depletion of Oma1 results in apoptotic resistance^26^. Although numerous studies have investigated MICOS, most studies have concentrated on MICOS structural features, crista formation and MICOS interactions with proteins. Therefore, little is known about the relationships between MICOS and diseases^27^.

In this study, we provided the first evidence showing that the Mic60 level declines with age and that its depletion induces TG accumulation in liver cells. In addition, we showed that vitamin D treatment rescued age-associated NAFLD by directly inducing Mic60 expression in a VDR-mediated manner. Collectively, these findings imply that MICOS 60 participates in the development of NAFLD and that the direct upregulation of Mic60 expression mediated via vitamin D supplementation may be a molecular mechanism underlying the effective prevention of NAFLD development, especially in elderly individuals.

## Materials and methods

### Materials

#### Animal studies

Male C57BL/6 mice aged 3 and 18 months were purchased from the Animal Facility of Aging Science, Korea Basic Science Institute (KBSI) Gwangju Center (Gwangju, Korea). After a week of adaptation, 3- or 18-month-old mice were randomly divided into two groups and fed the appropriate diet for 4 months (*n* = 10–12 per group). The control group was fed a standard chow diet (AIN-93G, Research Diets, NJ, USA) containing vitamin D_3_ (1000 IU/kg), and the vitamin D_3_-supplemented group was fed a standard chow diet enriched with vitamin D_3_ (20,000 IU/kg). All animal experiments were performed according to the guidelines of the Korean National Institutes of Health Animal Care and Use Committee (permit number: KCDC-032-20-2A).

### Isolation of primary hepatocytes

Primary hepatocytes were isolated from 10-week-old male C57BL/6 mice by collagenase (C5138, Merck, Darmstadt, Germany) perfusion. The cells were maintained in Medium 199 (M4530, Merck) supplemented with 10% FBS, 1% penicillin/streptomycin and 10 nM dexamethasone (D4902, Merck).

### Silencing of genes

Short interfering RNA (siRNA) was synthesized by Ambion (Life Technologies, CA, USA). The primer sequences against each siRNA were as follows: human *Immt* (5’-CACCCAAGCUUUAACCGCATT-3’ and 5’-UGCGGUUAAAGCUUGGGUGAA-3’), *LonP1* (5’-GAUUAUCGAGGUUAAAAAUTT-3’ and 5’-AUUUUUAACCUCGAUAAUCTT-3’), mouse *Immt* (5’-GGACAAUUCUGAGAUUGCATT-3’ and 5’-UGCAAUCUCAGAAUUGUCCAT-3’), mouse *LonP1* (5’-CCACACAAGGCAAGAUCCUCUGCUU-3’ and 5’-AAGCAGAGGAUCUUGCCUUGUGUGG-3’) and human *VDR* (5’-AGUUCAUUCUGACAGAUGATT-3’ and 5’-UCAUCUGUCAGAAUGAACUGG-3’). Transfection with the siRNAs was performed using Lipofectamine RNAiMAX (Invitrogen, Cambridge, UK) reagent for 24 h.

### Measurement of TG concentration

Liver tissues or HepG2 cells were homogenized in 1 ml of 5% NP-40 solution using a homogenizer for 30 s. The samples were slowly heated to 99 °C in a heat block for 5 min and then cooled to room temperature. The samples were centrifuged for 2 min at top speed with a microcentrifuge to remove insoluble material. For tissue obtained from animals, samples were diluted 10-fold with ddH_2_O, and for supernatants obtained from the cells, the samples were diluted 3-fold with ddH_2_O before TG analysis. Prepared samples were measured using a triglyceride assay kit (Abcam, Cambridge, UK).

### Immunofluorescence

Paraffin Section (5 μm) were deparaffinized and hydrated with xylene and ethanol. The sections were blocked with 2.5% normal horse serum at room temperature for 30 min and then incubated overnight at 4 °C with an anti-Mic60 antibody (Abcam). The sections were washed, incubated with Alexa Fluor-488 secondary antibody (Invitrogen, CA, USA) for 1 h and then washed with PBS. The sections were stained with DAPI (H-1200, Vector Laboratories, CA, USA) and observed under a fluorescence microscope. Images of the whole slide were captured using a confocal laser scanning microscope (FV3000-OSR, Olympus, Japan). The relative fluorescence intensity was measured using CellSens (Olympus) software.

### Electron microscopy

HepG2 cells were fixed with 2.5% glutaraldehyde in 0.1 M sodium cacodylate buffer pH 7.4 overnight. The samples were sequentially dehydrated in 50%, 70%, 90%, 95% and 100% ethanol. After embedding with Epon 812 (Merck, Darmstadt, Germany), 70-nm sections were sliced with an ultrathin microtome. The sections were stained with 1% uranyl acetate for 5 min, incubated with 1% lead citrate for 3 min, and observed with a transmission electron microscope (LIBRA-120, Carl Zeiss, Germany).

### Chromatin immunoprecipitation

ChIP analysis was performed according to the manufacturer’s instructions (Millipore, Darmstadt, Germany). Briefly, HepG2 cells were crosslinked with 1% formaldehyde for 10 min at room temperature and then quenched with 125 mM glycine. The cells were resuspended and sonicated in SDS lysis buffer. Lysates were incubated overnight at 4 °C with the following antibodies: anti-mouse IgG (Santa Cruz, Dallas, USA), anti-VDR (Santa Cruz) and anti-RXRα (Santa Cruz). Protein G agarose was added to form immunocomplexes, which were washed and subjected to elution. The samples were treated with RNase A and proteinase K. DNA was subsequently purified using PCR purification spin columns (QIAGEN, Hilden, Germany). The primers used to amplify the *Immt* promoter regions were designated R1 (−3986 to −3203), R2 (−3157 to −2323), R3 (−2312 to −1724), R4 (−1845 to −1159), R5 (−1179 to −550) and R6 (−574 to 115) from the transcription start site (TSS). The primers are listed in Supplementary Table 1.

### Promoter analysis with a luciferase reporter gene

To prepare human *Immt* reporter constructs, the promoter of human *Immt* was amplified by PCR. The PCR products were subcloned and inserted into the pGL3 promoter (E176A, Promega, WI, USA). The forward primers used for cloning were as follows: −3215/+114 (5’-ACCGGTACCCCTATGGTGGCCACTTTGAGTC-3’), −2244/+114 (5’- ACCGGTACCCCAAAATGCCGGGATATGAGC-3’), and 522/+114 (5’- ACCGGTACCCTATCCTTGTTGTGCCACTTGAC-3’). For all constructs, we used the same reverse primer (5’-ACCGCTAGCCGGTCACACCCGATAACTGAC-3’). Dual-luciferase assays were performed according to the manufacturer’s instructions (Promega, WI, USA). All experiments were carried out in triplicate and repeated at least 3 times.

### Statistical analysis

All results are expressed as the means ± standard errors of the means (SEMs). Statistical analysis was performed using GraphPad Prism software (GraphPad, CA, USA). Comparisons between two groups were performed with Student’s *t* test or the nonparametric Mann–Whitney *U* test. For multiple group comparisons, one-way analysis of variance (ANOVA) with Tukey’s post hoc test was performed to evaluate the significance of the differences. *P* values < 0.05 were considered to indicate statistical significance.

## Results

### Vitamin D supplementation restores the age-dependent reduction in liver Mic60 levels

**Fig. 1:**
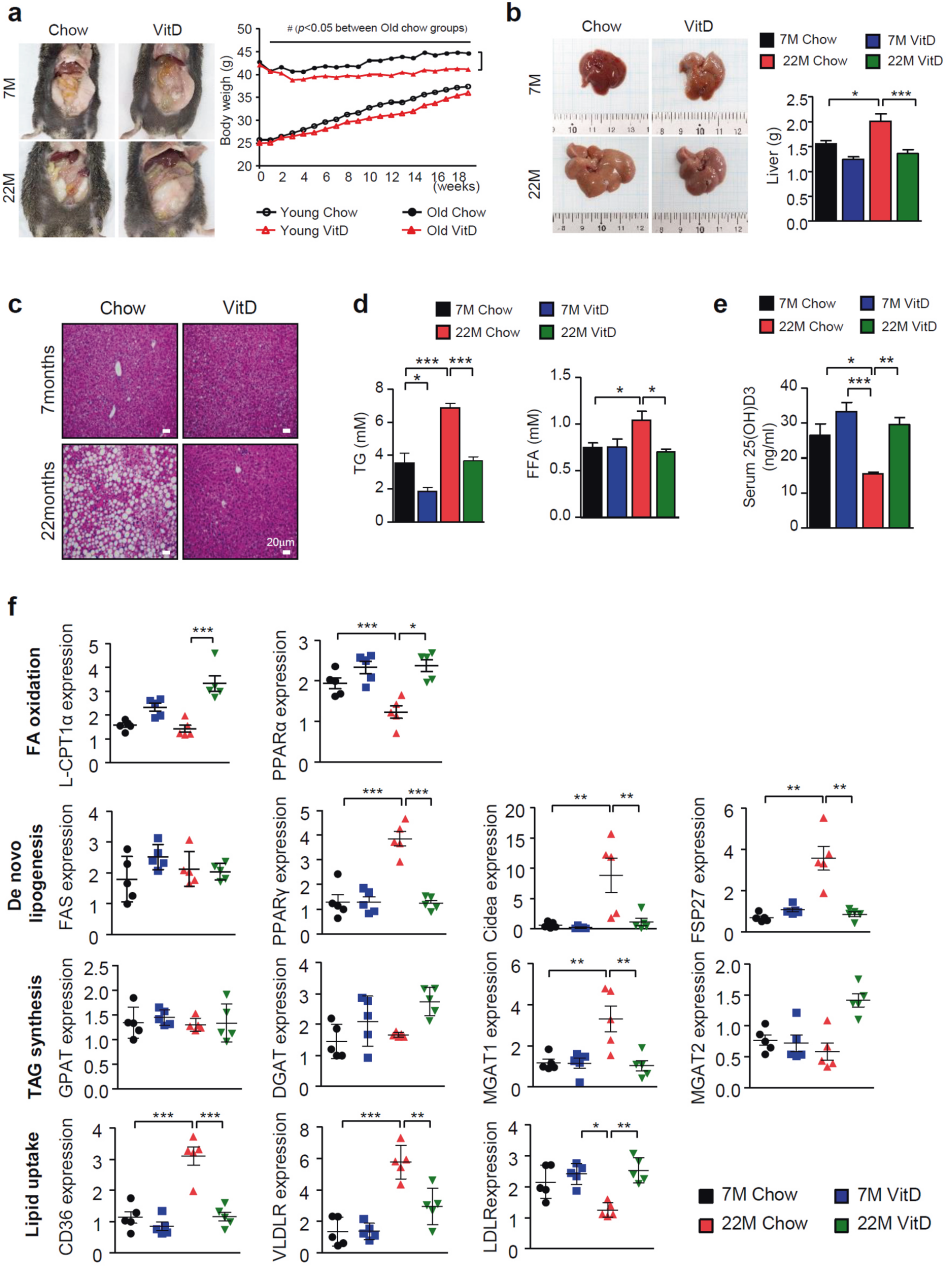
Vitamin D_3_ specifically prevents the development of NAFLD in aged mice. **a** Body weight changes in 3 month- or 18-month-old mice during the 18-week study period (*n* = 10–12). **b** The weight of livers extracted from mice as indicated. **c** Representative images of H&E stained liver tissue sections of 7- or 22-month-old mice. Scale bar = 30 μm. **d** Tissue TG and serum FFA levels were measured using ELISA kits according to the manufacturer’s instructions. Each value was normalized to that of the protein concentration (*n* = 5). **e** Serum vitamin D_3_ levels (*n* = 5). **f** Quantitative real-time PCR of genes related to lipid metabolism (*n* = 5). Statistical analyses were performed via one-way ANOVA with Tukey’s post hoc test for multiple comparisons; *p < 0.05, **p < 0.01, ***p < 0.001.

Vitamin D is thought to have beneficial functions in preventing NAFLD, but its effects remain controversial^28^. Moreover, most studies have investigated only the effect of vitamin D on NAFLD and have not explored the combination of vitamin D with multiple other risk factors, such as aging or mitochondrial dysfunction. In this study, we examined the precise molecular mechanisms through which vitamin D restores age-induced NAFLD, especially in relation to mitochondria. We first measured the liver mass and degree of lipid accumulation by hematoxylin and eosin (H&E)-stained samples from young (7-month-old) and aged (22-month-old) C57BL/6 mice. Vitamin D_3_ administration (20,000 IU/kg) led to significantly less weight gain compared with fed a control (1000 IU/kg) diet in aged mice (Fig. 1a). We observed a significant reduction in the abnormal expansion of the total liver mass and lipid accumulation within the liver only in the aged mice fed a vitamin D_3_-supplemented diet (Fig. 1b, c). In addition, the circulating FFA and hepatic TG levels were inversely correlated with serum 25(OH)D_3_ levels (Fig. 1d, e). Next, we measured gene expression patterns related to lipid homeostasis in the liver in samples extracted from each group of animals. Aging altered lipid metabolism to promote TG accumulation, and vitamin D_3_ supplementation led to the opposite results only in aged mice, corresponding with histological changes (Fig. 1f). In particular, we observed robust alterations in the expression levels of the following genes involved in DNL: peroxisome proliferator-activated receptor (PPAR)γ and cell death-inducing DNA fragmentation factor alpha-like effector A (CIDEA)^29^. Moreover, the expression levels of PPARα and carnitine palmitoyltransferase 1 (CPT-1), which are key regulators of β-oxidation, were reduced^30^. A marked increase in the expression levels of CD36, very low density lipoprotein receptor (VLDLR) and monoacylglycerol *O*-acyltransferase 1 (Mgat-1), a diacylglycerol (DAG) and TG synthesis catalase, was observed^31^.

**Fig. 2:**
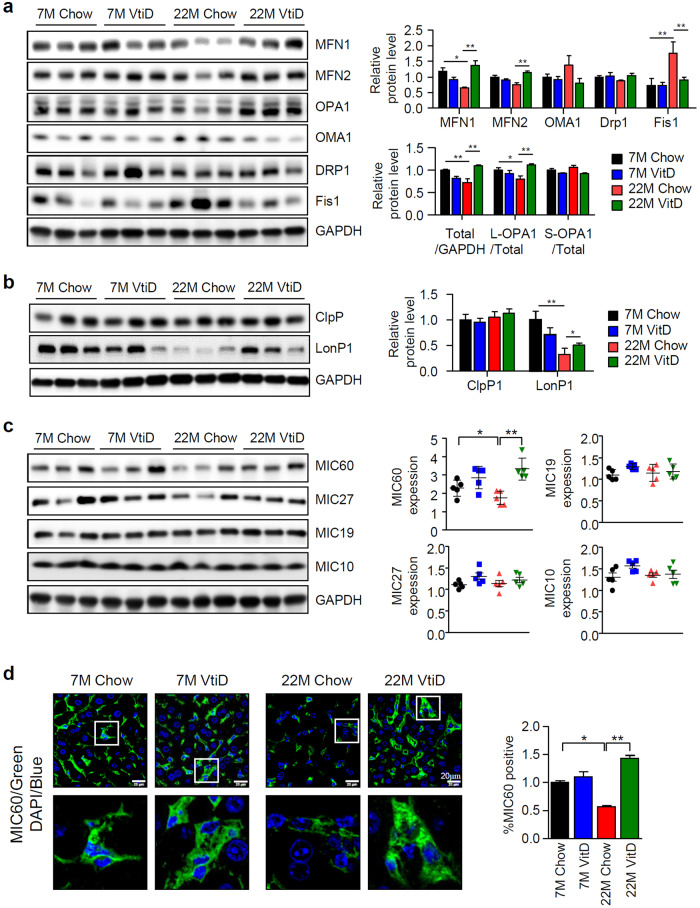
Alterations in MICOS and mitochondrial dynamics-associated protein levels in aged mice. Changes in the levels of proteins involved in either mitochondrial dynamics (**a**) or MQC (**b**) (*n* = 3). Relative values compared to the value of young mice fed a chow diet are presented as the means ± SEMs (*n* = 3). **c** Protein levels of MICOS subunits (left panel) and quantitative real-time PCR analysis of MICOS subunits (right panel, *n* = 5). **d** Immunofluorescence images of mouse liver tissue obtained using an anti-Mic60 antibody as indicated. Scale bars = 50 μm. Statistical analyses were performed via one-way ANOVA with Tukey’s post hoc test for multiple comparisons; *p < 0.05, **p < 0.01.

Aging and a decline in mitochondrial functions are closely associated^32^, and mitochondrial dynamics and MQC are important for the maintenance of optimal mitochondrial function. Therefore, we measured changes in the levels of proteins related to mitochondrial dynamics and MQC. In aged mice, the expression levels of proteins involved in OXPHOS (Supplementary Fig. 1) and mitochondrial fusion were reduced, and this decrease was reversed by vitamin D_3_ supplementation (Fig. 2a). Previously, Ryan Z. C. et al. reported similar effects of vitamin D, which increased the levels of mitochondrial fusion proteins, such as mitofusin1 (Mfn1) and Opa1, and reduced the levels of fission proteins, mitochondrial fission protein 1 (fis1) and Oma1 in skeletal muscle^33^. In agreement with the results shown in Fig. 1, the marked compensatory effects of vitamin D_3_ were observed only in the aged mice. Caseinolytic mitochondrial matrix peptidase proteolytic subunit (ClpP) and Lon protease 1 (LonP1) are representative MQC proteins located in the mitochondrial matrix^34^. We observed that only the level of LonP1 was specifically reduced in the aged mice and that the level was restored by vitamin D_3_ treatment (Fig. 2b).

MICOS is a relatively newly discovered mitochondrial protein complex; therefore, its role, especially in diseases, is largely unknown. To determine whether the level of MICOS was altered in our animal model system, we performed real-time PCR and Western blot analysis. Interestingly, we found that the expression of Mic60 was specifically reduced by aging, and the level was restored by vitamin D_3_ supplementation only in aged mice (Fig. 2c). To confirm these results, we performed immunofluorescence with an anti-Mic60 antibody and determined that the expression of Mic60 was inversely correlated with aging and restored by vitamin D_3_ supplementation (Fig. 2d). Considering these results, we demonstrated that aging, mitochondrial dysfunction, and NAFLD are closely related and that vitamin D_3_ supplementation is effective only when the vitamin D_3_ concentration in the blood is too low, as it is in aged mice. In particular, we observed a reduction in Mic60 and LonP1 levels due to aging and found that vitamin D_3_ supplementation restored these levels in aged mice.

### Depletion of Mic60 specifically induces TG accumulation in the liver

**Fig. 3:**
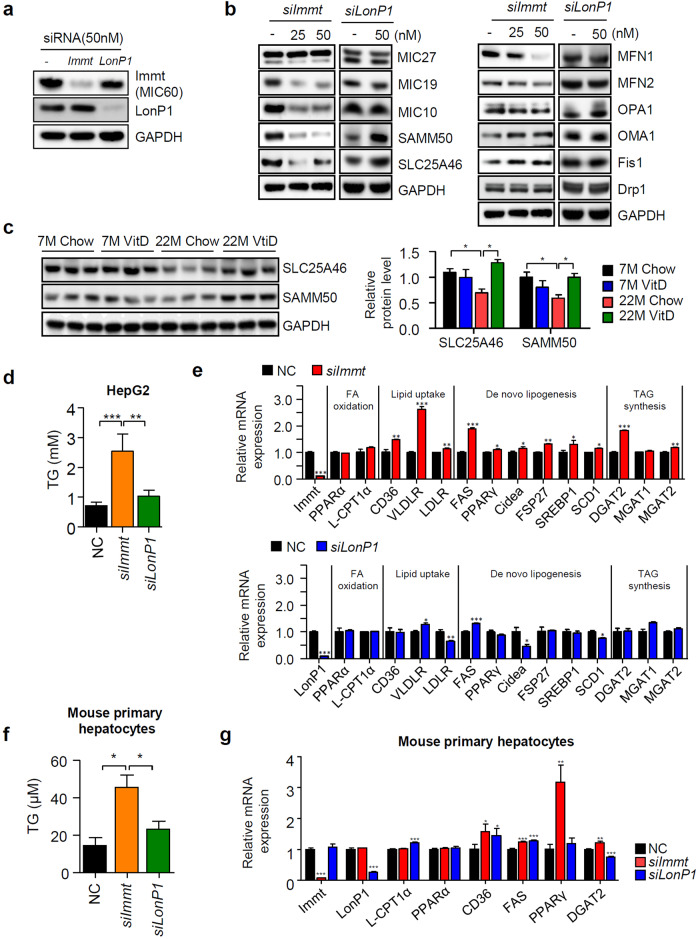
The knockdown of Mic60 specifically leads to TG accumulation. **a** Immunoblot analysis after transfection of individual siRNAs as indicated. **b** Alterations in the protein levels of MICOS proteins and proteins involved in mitochondrial dynamics after transfection of *siImmt* or *siLonP1* are shown. **c** Immunoblots showing the Sam50 and SLC25A46 levels in the liver tissue obtained from the animals as indicated (*n* = 3). **d** The TG level in HepG2 cells was measured with a TG assay kit following the manufacturer’s instruction (*n* = 3). **e** Genes related to lipid metabolism was measured by qPCR after the transfection of either *siImmt* or *siLonP1*. Statistical analyses were performed via one-way ANOVA with Tukey’s post hoc test for multiple comparisons; *p < 0.05, **p < 0.01, ***p < 0.001 (*n* = 3). Alterations in TG accumulation (**f**) and the expression of genes related to lipid metabolism (**g**) after the transfection of each siRNA into primary hepatocytes purified from mice were analyzed as described in Materials and Methods.

To examine whether the decrease in the Mic60 or Lonp1 levels with aging is related to fat accumulation in the liver, we first transfected HepG2 human hepatoma cells with siRNAs against *Immt* and *Lonp1*. In all cases, transfection of both siRNAs led to the specific depletion of individual genes (Fig. 3a), and the cellular ATP production level and mitochondrial membrane potential gradually decreased (Supplementary Fig. 2). Next, we examined whether the expression pattern of MICOS subunits is altered by depletion of Mic60 or LonP1. Significant changes were observed in MICOS subunits, except Mic27, after *Immt* silencing, but no profound reduction in MICOS subunit levels was observed after *siLonP1* silencing (Fig. 3b left panel). In addition, we observed the influences of depleting either Mic60 or Lonp1 on mitochondrial dynamics. We found that the silencing of Mic60 led to upregulated Fis1, Oma1 and dynamin-related protein 1 (Drp1) expression, downregulated Mfn1 and Mfn2 expression and decreased expression of Opa1, and these effects induced changes in the direction toward mitochondrial fragmentation (Fig. 3b right panel). In contrast, depletion of LonP1 did not lead to any significant alteration in the levels of proteins involved in mitochondrial dynamics.

Interestingly, we observed opposite changes in the Sam50 and SLC25A46 levels after the depletion of Mic60 or LonP1. We therefore examined the amount of both proteins in our animal models. As shown in Fig. 3c, the protein expression of Sam50 and SLC25A46 was reduced in aged mice and restored by vitamin D_3_, similar to the Mic60 level. Both Sam50 and SLC25A46 interact with Mic60^21,24^ but not with LonP1. Therefore, the reduction in the Sam50 and SLC25A46 levels in aged mice seemed to be related to the reduction in Mic60 levels observed in aged mice.

To determine whether these alterations in mitochondria caused by the silencing of specific RNAs are related to fat accumulation in the liver, we measured the amount of TG after the silencing of *Immt* or *Lonp1*. Interestingly, we found that only the loss of Mic60 specifically increased TG accumulation in the liver. In addition, we determined that the levels of the genes involved in DNL, lipid uptake and TG synthesis were greatly increased by the loss of Mic60, similar to the observations in our animal model. These outcomes were evident only in the absence of Mic60 but not in the absence of LonP1 (Fig. 3e). To verify the specific accumulation of TG by *siImmt* transfection, we isolated primary hepatocytes from the livers of 10-week-old male mice. As observed in the HepG2 cell line, TG accumulated in primary hepatocyte cells only after *siImmt* transfection, and the expression of genes related to lipid accumulation was elevated only in the absence of Mic60 (Fig. 3f, g). Based on these results, we hypothesize the existence of a specific relationship between the loss of Mic60 and TG accumulation in liver cells.

### Vitamin D restores mitochondrial function and prevents TG accumulation after loss of Mic60

**Fig. 4:**
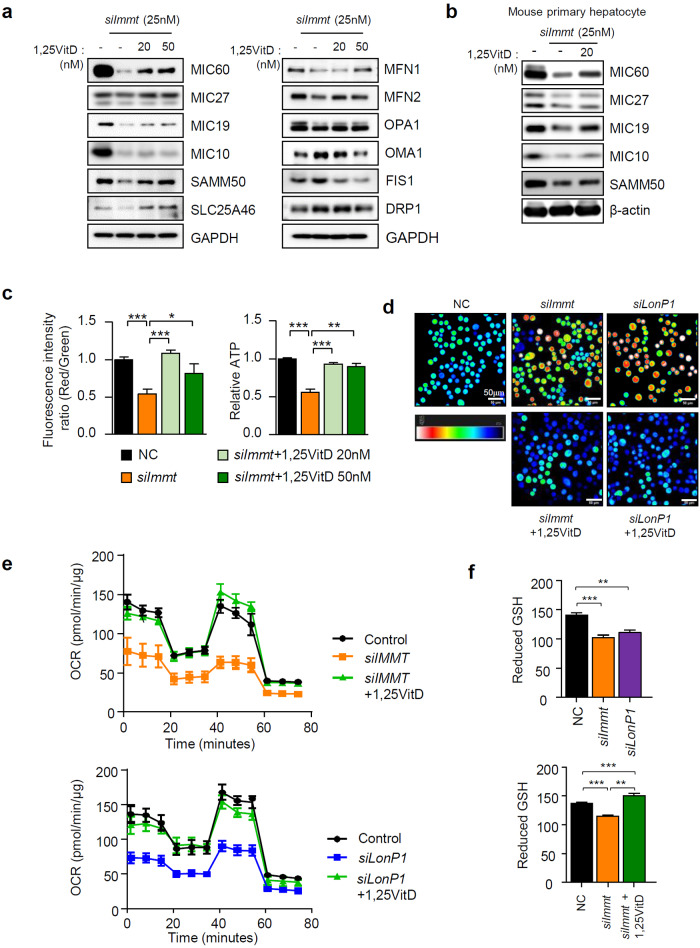
Mic60 expression is correlated with Vitamin D level. HepG2 cells (**a**) or mouse primary hepatocyte cells (**b**) were treated with 1,25VitD_3_ 24 h after *siImmt* transfection. Immunoblot analyses were performed using antibodies as indicated. **c** The mitochondrial membrane potential was measured with a JC-1 assay kit (Abcam, left panel), and cellular ATP amounts (right panel) were measured with ATP colorimetric/fluorometric assay kit (Abcam), according to the manufacturer’s instructions. **d** Intracellular ROS were quantified by 2’,7’-dichlorofluorescin after incubation for 20 min in serum-free media containing 10 µM dichlorofluorescin diacetate (DCF-DA, Invitrogen). **e** The oxygen consumption rate (OCR) in live cells was measured using Seahorse XF HS Mini (Agilent Technologies, CA, USA) according to the manufacturer’s protocol. The OCR levels were normalized to the amount of protein in each sample. **f** The amount of reduced GSH was determined using a colorimetric assay kit (EZ-glutathione assay kit, DG-GLU200, DogenBio, Seoul, Korea) according to the manufacturer’s instructions (reduced GSH = GSH-2GSSG).

**Fig. 5:**
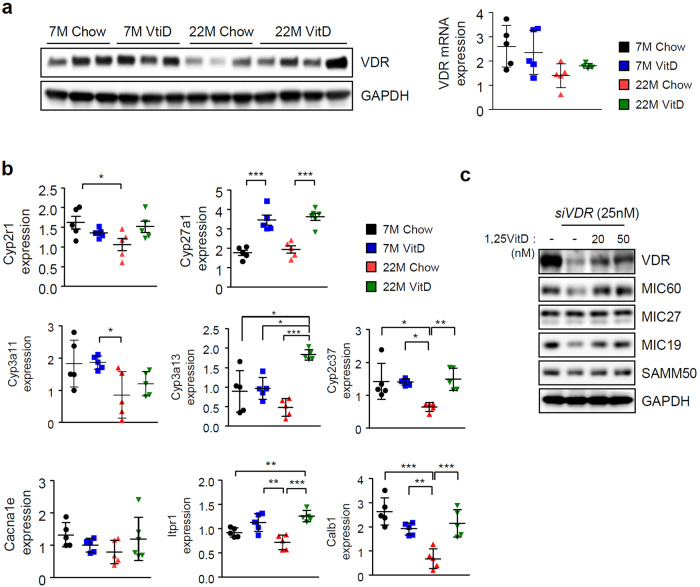
Alterations in the expression levels of VDR and its related genes in aged mice. **a** Changes in VDR levels in aged mice with or without vitamin D_3_ supplementation. Left panel: Western blot; right panel: qPCR (*n* = 5). **b** Alterations in the expression patterns of representative genes involved in VDR-mediated transcriptional networks in each animal groups (*n* = 5). **c** VDR-dependent alterations in the levels of MICOS-related proteins in HepG2 cells. Western blotting was performed 24 h after *siVDR* transfection.

In aged mice, vitamin D_3_ supplementation effectively prevented the reduction in Mic60 levels. To test whether vitamin D_3_ affects Mic60 expression in cell models, we treated cells with 1,25VitD_3_ after silencing *Immt*. Although *siImmt* transfection completely depleted Mic60 in cells, 1,25VitD_3_ treatment somewhat restored the levels of Mic60 protein and other MICOS components (Fig. 4a, left panel). Notably, mitochondrial fusion proteins were also restored by vitamin D (Fig. 4a right panel)_._ To confirm the vitamin D-dependent induction of Mic60, we silenced *siImmt* using mouse primary hepatocyte cells in the presence or absence of vitamin D and observed the same effects in HepG2 cells (Fig. 4b). Together with the elevation in the levels of Mic60 observed after vitamin D treatment, the mitochondrial membrane potential and cellular ATP levels were recovered (Fig. 4c). In addition, intracellular ROS were increased by both *siImmt* and *siLonP1* (Fig. 4d), and this elevation was accompanied by a reduction in the oxygen consumption rate and a reduction in the GSH levels (Fig. 4e, f). These effects were restored by vitamin D treatment. Next, we tested whether VDR was truly reduced in aged mice and recovered by vitamin D_3_ treatment. The alteration of both the mRNA and protein levels of VDR in aged mice was the same as that found for Mic60 (Fig. 5a). We also investigated the changes in the VDR-mediated transcriptional network by examining gene expression patterns that are typically regulated by VDR, for example, genes involved in vitamin D metabolism, detoxification in the liver and calcium homeostasis. The expression levels of these genes were downregulated in aged mice, and these levels and VDR expression levels were restored after vitamin D_3_ feeding (Fig. 5b). To check the relationship between vitamin D and the MICOS system, we investigated the alterations in MICOS subunit expression levels after transfection with *siVDR* and found that, as expected, the Mic60 levels were reduced by loss of VDR and recovered by 1,25VitD_3_ treatment (Fig. 5c).

**Fig. 6:**
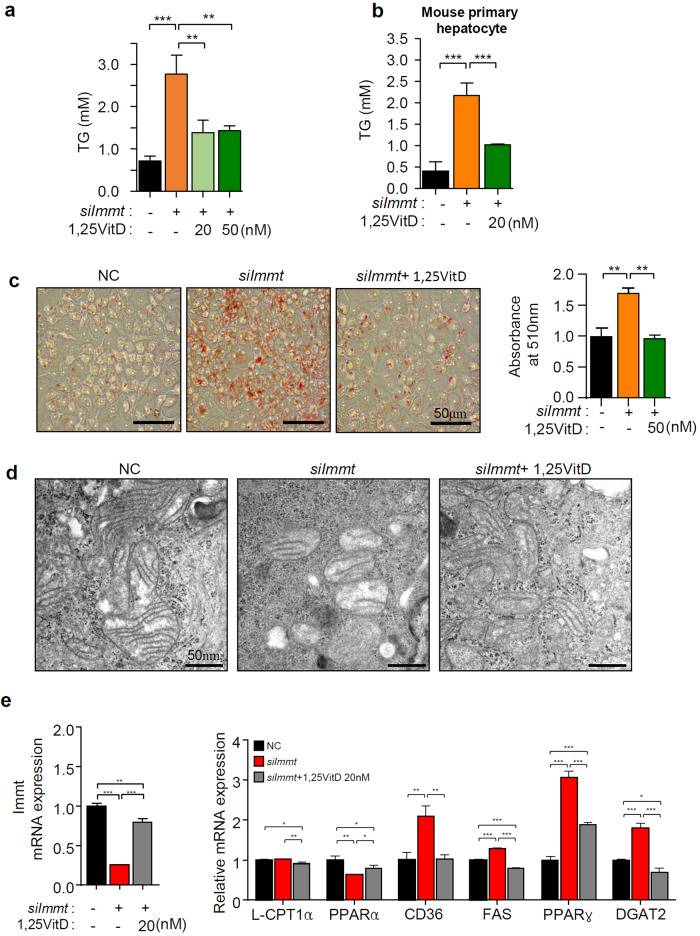
Vitamin D_3_ supplementation prevents TG accumulation by restoring the Mic60 level. TG levels in either HepG2 cells (**a**) or mouse primary hepatocyte cells (**b**) (*n* = 3). **c** Cellular lipid droplets were visualized by Oil red O staining (left panel) and quantified (right panel) (*n* = 3). **d** Electron microscopy images of HepG2 cells after the indicated treatment. Scale bars = 50 nm. **e** Recovery of the Mic60 level (left panel) and alterations in the levels of representative genes involved in lipid metabolism (right panel) in *siImmt*-transfected-mouse primary hepatocyte cells by 1,25VitD_3_ treatment.

We also observed that 1,25VitD_3_ treatment effectively prevented TG accumulation caused by a decrease in the Mic60 level in both HepG2 and mouse primary hepatocytes (Fig. 6a, b). To verify the fat accumulation specifically induced by the loss of Mic60 and the prevention of this outcome after 1,25VitD_3_ treatment, we performed an oil red O staining assay after *siImmt* transfection in the presence and absence of 1,25VitD_3_. We observed significant fat accumulation after *siImmt* transfection and a reduction in the number of lipid droplets after 1,25VitD_3_ treatment (Fig. 6c). Recently, Stephan T. et al. showed that after depletion of *Immt* in HeLa cells, normal crista formation was largely disrupted, and most cristae formed scattered small ladder-like shapes^35^. We next examined whether vitamin D can restore mitochondrial morphology that had been disrupted by the depletion of Mic60. We observed mitochondrial shapes through an electron microscope 24 h after *siImmt* treatment in the presence or absence of 50 nM 1,25VitD_3_. According to a decrease in the Mic60 level, we found that the number of normal mitochondria was reduced, and 1,25VitD_3_ treatment restored the normal morphology of mitochondria (Fig. 6d). To confirm the specific lipid accumulation induced by loss of Mic60, we knocked down Mic60 expression levels in primary hepatocytes. The expression of Mic60 was greatly reduced by the transfection of *siImmt* but significantly restored by vitamin D treatment. Concomitantly, the gene expression patterns were changed in the direction of lipid accumulation, and this alteration was effectively prevented by vitamin D treatment, as observed in HepG2 cells (Fig. 6e).

### Mic60 is correlated with cellular senescence

**Fig. 7:**
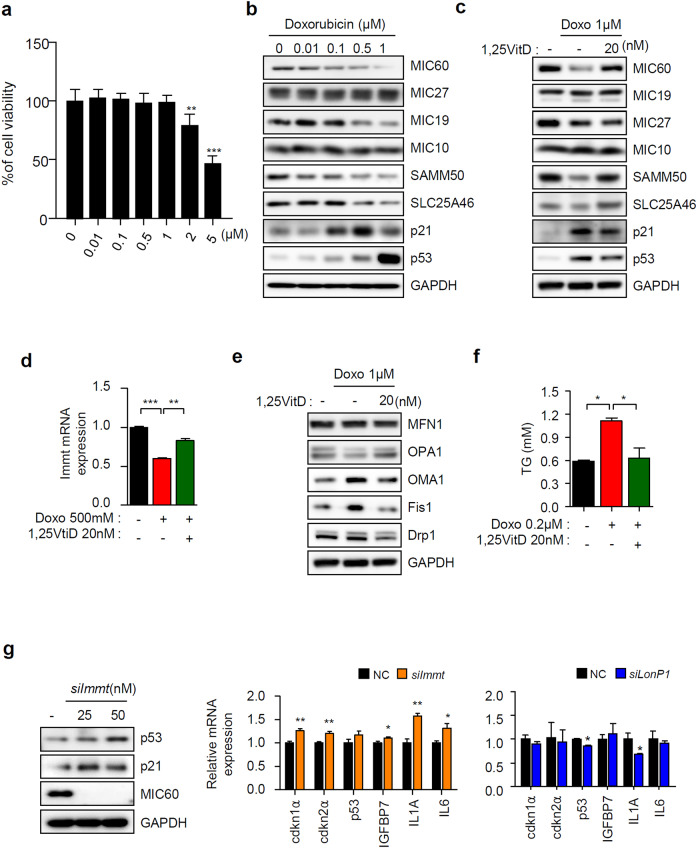
Induction of cellular senescence triggered a reduction in the Mic60 level. **a** Measurement of the cellular cytotoxicity of doxorubicin at different concentrations for 24 h using an MTT assay. **b** The MICOS protein levels were altered by increasing the concentration of doxorubicin. **c** Western blot analysis after induction of senescence in the presence or absence of 20 nM 1,25VitD_3_. **d** Measurement of *Immt* expression by qRT‒PCR (*n* = 5). **e** Analysis of the effects of Dox treatment on the mitochondrial dynamics, as determined by immunoblotting, as indicated. **f** Changes in TG accumulation by doxorubicin in the presence or absence of 1,25VitD_3_. **g** The silencing of *Immt*, but not *LonP1*, promoted the expression of cell senescence markers (*n* = 3).

To confirm that aging and a reduction in the Mic60 levels were related to each other, we established a cell model similar to that of the aging animal model by treating HepG2 cells with doxorubicin (Dox) as indicated^36^. The cell viability gradually decreased after 2 µM Dox treatment; therefore, we used Dox at concentrations lower than 1 µM (Fig. 7a). By increasing the concentration of Dox, the levels of representative senescence marker proteins, such as p21 and p53, were greatly increased. In contrast, the level of Mic60 gradually decreased, as observed in our animal models (Fig. 7b). We next examined the effects of vitamin D after the induction of cellular senescence. The levels of a senescence marker protein and Mic60 were inversely correlated, and vitamin D specifically upregulated the expression of Mic60, which is consistent with observations in the animal model (Fig. 7c). To verify the recovery of Mic60 induced by vitamin D treatment, we measured *Immt* expression after Dox treatment in the absence or presence of 1,25VitD_3_ and confirmed that vitamin D specifically upregulated *Immt* expression (Fig. 7d). We also measured the alterations in the levels of proteins involved in mitochondrial dynamics after Dox treatment and observed changes similar to those observed after the depletion of Mic60. A decrease in mitochondrial fusion and an increase in fission were observed, and these effects were reversed by 1,25VitD_3_ treatment (Fig. 7e). To verify that cellular senescence and the concomitant reduction in Mic60 were related to lipid accumulation, we measured the TG contents in Dox-treated cells in the presence or absence of vitamin D. As observed in aged mice, the induction of cellular senescence promoted TG accumulation, and this promotion was effectively prevented by vitamin D (Fig. 7f). Next, we examined whether silencing *Immt* triggered cellular senescence. As shown in Fig. 7g, the loss of Mic60 significantly induced several senescence markers, including p53, p21 and interleukin 1 A, but these effects were not observed in the absence of LonP1. Based on these experiments, we confirmed that the Mic60 level markedly decreased with aging and cellular senescence and that vitamin D specifically restored the senescence-dependent reduction in the Mic60 levels. In addition, an absence or reduction in Mic60 levels may specifically trigger cellular senescence and aging.

### Vitamin D regulates Immt expression by directly binding VDR-RXR to the Immt promoter

**Fig. 8:**
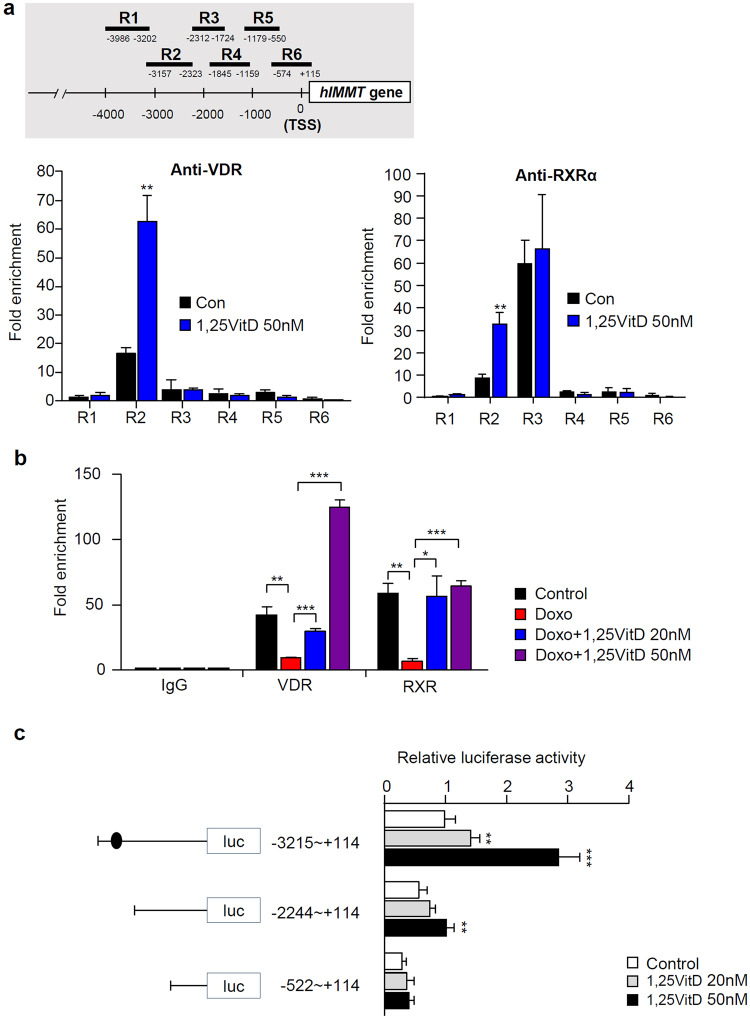
VDR-RXR upregulated *Immt* expression by directly binding to the *Immt* gene. **a** Chromatin immunoprecipitation (ChIP) assay was performed using either anti-VDR or anti-RXR antibodies as described in Materials and Methods. **b** ChIP of control vehicle or 500 µM doxorubicin treatment in HepG2 cells in combination with 20 nM or 50 nM 1,25VitD_3_ using the R2 region of the *Immt* promoter. The recruitment of VDR or RXRα to the position R2 ranging from −3157 to −2323 was the representative value for comparisons to the IgG control (three sets per group). **c** Luciferase reporter gene analysis of whole *Immt* (−3215 to +114) or deletion mutants from −2244 (−2244 to +114) and −522 (−522 to +114) (*n* = 3). Statistical analyses were performed via one-way ANOVA with Tukey’s post hoc test for multiple comparisons; *p < 0.05, **p < 0.01, ***p < 0.001.

The binding of vitamin D to VDR, together with its heterodimeric receptor retinoid X receptor (RXR), usually triggers conformational changes, which enable the recognition of vitamin D-responsive elements (VDREs) on vitamin D target genes. VDRE usually does not comprise conserved linear sequences; it is more three-dimensional. Additionally, variant VDRE sequences may influence unique complexes carrying VDR-RXR^37^. To test whether the elevation of Mic60 levels by vitamin D exerts indirect effects, such as alterations in Ca^2+^ homeostasis or oxidative stress, or whether vitamin D directly regulates Mic60 levels by VDR binding to its promoter, we performed chromatin immunoprecipitation (ChIP) using anti-VDR and anti-RXR antibodies. First, we searched for possible VDREs or retinoid X receptor responsive elements (RXREs) throughout the *Immt* gene, from the 5´ intron to the 3´ tail sequence, using NCBI BLAST suite SRA (SRX100497; HepG2_IP RXR) and found six candidate regions, designated R1 to R6. We assessed the recruitment of VDR and RXRα to all these candidate VDRE or RXRE sites in the regulatory region 5´ upstream in the *Immt* promoter and found one specific high-affinity VDR-RXR-binding site located in the region spanning positions −3157 to −2323 from the TSS, designated R2. VDR and RXR binding at this site were dramatically increased in the presence of vitamin D, indicating that VDR binding to the *Immt* promoter is ligand-dependent (Fig. 8a). To investigate whether VDR binding at this site is changed in cellular senescence conditions similar to aging mice, we performed ChIP of HepG2 cells after Dox treatment. The binding of both VDR and RXRα to this VDRE was dramatically reduced by Dox treatment but was very strong in the presence of the VDR-RXR ligand 1,25VitD_3_ (Fig. 8b). These results reveal, for the first time, that VDR-RXR directly binds to *Immt* in a region ranging from position −3157 to −2323 and thereby specifically upregulates *Immt* expression mediated by vitamin D. To confirm that VDR regulates the *Immt* gene by directly binding to the promoter in the R2 region, we prepared reporter gene constructs containing different lengths of *Immt* promoter regions, the whole *Immt* promoter (−3215 to +114) and two deletion mutants from −2244 (−2244 to +114) and −522 (−522 to +114). Only one reporter gene construct containing the R2 region (−3257 to −2323) induced luciferase activity by increasing the concentration of vitamin D (Fig. 8c), indicating that VDR could bind to the R2 region in a ligand-dependent manner.

## Discussion

The aging population is rapidly increasing worldwide; therefore, interest in research on aging-associated diseases, such as Parkinson’s disease and metabolic syndrome, has been increasing. The prevalence of NAFLD is also closely related to aging. In this study, we showed, for the first time, that depletion of Mic60 was directly associated with age-induced NAFLD development. In this work, we report multiple interesting facts. (i) Aging triggered a specific reduction in Mic60 levels. (ii) Depletion of Mic60 disrupted lipid homeostasis in liver cells, which directly led to TG accumulation. (iii) Vitamin D_3_ prevented lipid accumulation in aged mice and in senescence-induced HepG2 cells, which coincided with the recovery of the Mic60 level. (iv) The loss of Mic60 itself disrupted mitochondrial dynamics and triggered cellular senescence. (v) Vitamin D_3_ upregulated Mic60 expression via the recruitment of VDR-RXRα to the promoter of the *Immt* gene.

Mitochondrial dysfunction and concomitant elevation of oxidative stress are associated with numerous diseases, including chronic diseases and even cancer^38,39^. Although MICOS is important for crista formation and related mitochondrial functions, most studies on MICOS have thus far focused on MICOS structural effects. However, studies on the relationship between MICOS and human diseases have recently started to gain interest.

To date, representative studies on the association between MICOS and chronic diseases include the multiple reports. Baseler W.A. et al. reported a reduction in the Mic60 levels in type 1 diabetic hearts^40^, and Thapa D. et al. showed that transgenic cardiac-specific overexpression of Mic60 ameliorates diabetic cardiomyopathy^41^. Additionally, Guarani V. et al. demonstrated that the Mic13-null mutant is critical to early onset fetal mitochondrial encephalopathy with liver diseases^42^ and that Mic26 is overexpressed in the human diabetic heart^43^.

Our findings clearly show, for the first time, that the age-dependent reduction in the Mic60 level or depletion of Mic60 specifically induces TG accumulation, a key characteristic of NAFLD (Fig. 3). We hypothesize that lipid accumulation is the result of complex and diverse changes induced by the loss of Mic60-associated mitochondrial dysfunction, which is closely interrelated with aging. Mitochondrial dysfunction triggers an aberrant increase in hepatic gluconeogenesis and insulin resistance^44^. However, the increased levels of acetyl-CoA, as an excess glucose metabolite, cannot be used in the Krebs cycle for the generation of ATP due to a decrease in the mitochondrial membrane potential and a reduction in β-oxidation in mitochondria. Therefore, the excess acetyl-CoA is used in lipogenesis^45^. In addition, it is well known that CD36 is considered the major contributor to lipid uptake and TG accumulation in NAFLD. We showed a dramatic increase in CD36 expression in aged mice and *siImmt*-transfected cells (Figs. 1f and 3d, g). We hypothesize that the induction of peroxisome proliferator-activated receptor γ (PPARγ) with aging and the concomitant loss of Mic60 are likely involved in the increase in CD36 because CD36 is a well-known target gene of PPARγ. In addition, CD36 and mitochondrial β oxidation are closely related^46^. Chen Y. et al. reported that mitochondrial ROS are correlated with CD36 levels in a diet-induced murine model of atherosclerosis^47^. We therefore hypothesized that the accumulation of visceral fat in aged mice consequently elevates the serum levels of FFAs and increases lipid uptake in hepatocytes, which leads to a reduction in Mic60 levels, decreases in mitochondrial functions and sequential induction of multiple lipogenic transcription factors and their target genes, such as CD36. Together, these factors create a vicious cycle that worsens NAFLD in aged mice.

Although deletion of both Mic60 and LonP1 leads to reductions in mitochondrial functions and the elevation of oxidative stresses (Fig. 4d–f), loss of Mic60 induced mitochondrial dysfunction more severely than loss of LonP1. Nevertheless, we considered reasons for why only the depletion of Mic60 specifically led to TG accumulation in liver cells. These findings may imply the possibility that an additional mechanism rather than a reduction in overall mitochondrial function is involved in TG accumulation. We observed specific reductions not only in the level of Mic19, one of the MIC60-Mic19-Mic25 subunits, but also in the levels of sam50, SLC25A46, Opa1 and Mfn1 and an increase in the levels of Oma1 and Fis1 (Fig. 3b). These outcomes were observed only in the absence of Mic60, not in the absence of LonP1. Recently, Tang J. et al. reported that the Sam50-Mic19-Mic60 interaction is important to the connection between the OM and the IM and is involved in normal crista shape formation^48^. In addition, they found that Oma1 disrupted this membranous connection by cleaving Mic19. Recently, using liver-specific Sam50-knockout mice, Chen L. et al. reported that the depletion of Sam50 induced liver inflammation and liver injury^49^. In addition, the *SAMM50* polymorphism has been reported to be associated with NAFLD^50^. Considering these studies, we hypothesize that Mic60 may play a central role not only in mitochondrial crista formation and the maintenance of mitochondrial dynamics but also in the connection between mitochondrial function and the environment outside organelles via its interactions with multiple proteins. Therefore, Mic60 may influence the development of NAFLD. Although Lonp1 is important for maintaining overall mitochondrial function, we found that the loss of Lonp1 did not induce TG accumulation. We suggest that LonP1 did not induce fat accumulation because it did not induce a change in the mitochondrial structure and thus exerted no overall influence on the connection between the OM and IM. This occurs even though Lonp1 plays a central role in MQC and is important to maintaining mitochondrial function. Furthermore, we speculate that only the loss of Mic60 is involved in lipid accumulation is because only the loss of Mic60, but not LonP1, specifically induced cellular senescence (Fig. 7g). The induction of cellular senescence by reductions in the levels of Mic60 could accelerate aging and thus cause a greater reduction in Mic60, which more vigorously induces circumstances that may facilitate NAFLD. However, additional studies are still needed to determine the precise molecular mechanisms through which the depletion of Mic60 may promote NAFLD.

Many reports, including those of Roth C. L. et al., have suggested the therapeutic effects of vitamin D on NAFLD^51^. In the current study, vitamin D effectively prevented TG accumulation only in aged mice. Similarly, we observed a preventive effect of vitamin D_3_ on pancreatic dysfunction and hyperinsulinemia defects only in aged mice^52^. Pines A. et al. showed that the beneficial effects of vitamin D_3_ supplementation were greater in aged men than in younger men^53^. The optimal vitamin D_3_ concentration varies considerably, and the normal concentration of vitamin D_3_ in human serum is typically considered to be 25–80 ng/mL^10^. In our mouse model, the serum vitamin D_3_ level was insufficient only in aged mice fed a normal chow diet, and this level was restored in aged mice fed a diet supplemented with vitamin D_3_ (Fig. 1e). Interestingly, we found that vitamin D_3_ restored the expression of Mic60 only in aged mice fed a diet supplemented with vitamin D_3_ (Fig. 2c, d) and in senescence-induced HepG2 cells (Fig. 7c). Considering these observations, we suggest that it is important to maintain sufficient serum vitamin D and VDR levels in hepatocytes in aged mice to preserve sufficient Mic60 levels in the liver and thus prevent mitochondrial dysfunction and subsequent age-related NAFLD development. In addition, we showed that VDR-RXR bound directly to *Immt* at the promoter region (−3157 to −2323) to regulate its expression in a ligand-dependent manner via ChIP and reporter gene analysis (Fig. 8a, c). We found one more RXR binding site, R3 (−2312 to −1724), to which VDR did not bind. However, RXR binding to the R3 region of the *Immt* promoter was ligand-independent. Even RXR bound at the R3 site may recruit VDR, but this binding is nonspecific and ligand-independent, and therefore, the amount may be considered negligible. Based on these results, we hypothesize that vitamin D upregulates Mic60 expression via direct binding to its promoter only at the R2 region (−3157 to −2323) in aged mice fed a diet supplemented with sufficient vitamin D.

In conclusion, in this study, we first demonstrated that the depletion of Mic60 is related to the development of age-induced NAFLD and that vitamin D can prevent NAFLD by upregulating Mic60 expression in a VDR-RXR-dependent manner. Further studies are needed to determine whether the loss of Mic60 is related to the development of age-dependent NAFLD in humans.

### Supplementary information

Supplementary Information

Supplementary Figure for reviewer only

## References

[1] Maurice J, Manousou P (2018). Non-alcoholic fatty liver disease. Clin. Med..

[2] Ipsen DH, Lykkesfeldt J, Tveden-Nyborg P (2018). Molecular mechanisms of hepatic lipid accumulation in non-alcoholic fatty liver disease. Cell Mol. Life Sci..

[3] Li Y, Adeniji NT, Fan W, Kunimoto K, Torok NJ (2022). Non-alcoholic Fatty Liver Disease and Liver Fibrosis during Aging. Aging Dis.

[4] Gong Z, Tas E, Yakar S, Muzumdar R (2017). Hepatic lipid metabolism and non-alcoholic fatty liver disease in aging. Mol. Cell Endocrinol..

[5] Barzilai N, Huffman DM, Muzumdar RH, Bartke A (2012). The critical role of metabolic pathways in aging. Diabetes.

[6] Berryman DE, Christiansen JS, Johannsson G, Thorner MO, Kopchick JJ (2008). Role of the GH/IGF-1 axis in lifespan and healthspan: lessons from animal models. Growth Horm IGF Res.

[7] Kamagate A (2008). FoxO1 mediates insulin-dependent regulation of hepatic VLDL production in mice. J. Clin. Investig..

[8] Haussler MR (2013). Molecular mechanisms of vitamin D action. Calcif Tissue Int.

[9] Berridge, M. J. Vitamin D, reactive oxygen species and calcium signalling in ageing and disease. *Philos. Trans. R Soc Lond B Biol. Sci.* **371**, 10.1098/rstb.2015.0434 (2016). 10.1098/rstb.2015.0434 PMC4938033 27377727

[10] Kennel KA, Drake MT, Hurley DL (2010). Vitamin D deficiency in adults: when to test and how to treat. Mayo Clin. Proc..

[11] Eliades M, Spyrou E (2015). Vitamin D: a new player in non-alcoholic fatty liver disease?. World J. Gastroenterol..

[12] Jaruvongvanich V, Ahuja W, Sanguankeo A, Wijarnpreecha K, Upala S (2017). Vitamin D and histologic severity of nonalcoholic fatty liver disease: A systematic review and meta-analysis. Dig Liver Dis..

[13] Sebastian D, Palacin M, Zorzano A (2017). Mitochondrial dynamics: coupling mitochondrial fitness with healthy aging. Trends Mol. Med..

[14] Wiedemann N, Pfanner N (2017). Mitochondrial machineries for protein import and assembly. Annu. Rev. Biochem..

[15] Yi HS, Chang JY, Shong M (2018). The mitochondrial unfolded protein response and mitohormesis: a perspective on metabolic diseases. J. Mol. Endocrinol..

[16] Li R, Toan S, Zhou H (2020). Role of mitochondrial quality control in the pathogenesis of nonalcoholic fatty liver disease. Aging.

[17] Takeichi Y (2021). Non-alcoholic fatty liver disease in mice with hepatocyte-specific deletion of mitochondrial fission factor. Diabetologia.

[18] Cogliati S, Enriquez JA, Scorrano L (2016). Mitochondrial Cristae: Where Beauty Meets Functionality. Trends Biochem. Sci..

[19] Harner M (2011). The mitochondrial contact site complex, a determinant of mitochondrial architecture. EMBO J..

[20] van der Laan M, Horvath SE, Pfanner N (2016). Mitochondrial contact site and cristae organizing system. Curr. Opin. Cell Biol..

[21] Colina-Tenorio L, Horten P, Pfanner N, Rampelt H (2020). Shaping the mitochondrial inner membrane in health and disease. J. Intern. Med..

[22] Tsai PI (2018). PINK1 Phosphorylates MIC60/Mitofilin to Control Structural Plasticity of Mitochondrial Crista Junctions. Mol. Cell.

[23] Patten DA (2014). OPA1-dependent cristae modulation is essential for cellular adaptation to metabolic demand. EMBO J..

[24] Janer A (2016). SLC25A46 is required for mitochondrial lipid homeostasis and cristae maintenance and is responsible for Leigh syndrome. EMBO Mol. Med..

[25] Jiang X, Jiang H, Shen Z, Wang X (2014). Activation of mitochondrial protease OMA1 by Bax and Bak promotes cytochrome c release during apoptosis. Proc. Natl Acad. Sci. USA.

[26] Viana MP, Levytskyy RM, Anand R, Reichert AS, Khalimonchuk O (2021). Protease OMA1 modulates mitochondrial bioenergetics and ultrastructure through dynamic association with MICOS complex. iScience.

[27] Eramo MJ, Lisnyak V, Formosa LE, Ryan MT (2020). The ‘mitochondrial contact site and cristae organising system’ (MICOS) in health and human disease. J. Biochem..

[28] Keane, J. T., Elangovan, H., Stokes, R. A. & Gunton, J. E. Vitamin D and the Liver-Correlation or Cause? *Nutrients* **10**, 10.3390/nu10040496 (2018). 10.3390/nu10040496 PMC5946281 29659559

[29] Zhou L (2012). Cidea promotes hepatic steatosis by sensing dietary fatty acids. Hepatology.

[30] Bechmann LP (2012). The interaction of hepatic lipid and glucose metabolism in liver diseases. J. Hepatol..

[31] Lee YJ, Kim JW (2017). Monoacylglycerol O-acyltransferase 1 (MGAT1) localizes to the ER and lipid droplets promoting triacylglycerol synthesis. BMB Rep..

[32] Janikiewicz J (2018). Mitochondria-associated membranes in aging and senescence: structure, function, and dynamics. Cell Death Dis..

[33] Ryan ZC (2016). 1alpha,25-Dihydroxyvitamin D3 Regulates Mitochondrial Oxygen Consumption and Dynamics in Human Skeletal Muscle Cells. J. Biol. Chem..

[34] Quiros PM, Langer T, Lopez-Otin C (2015). New roles for mitochondrial proteases in health, ageing and disease. Nat. Rev. Mol. Cell Biol..

[35] Stephan T (2020). MICOS assembly controls mitochondrial inner membrane remodeling and crista junction redistribution to mediate cristae formation. EMBO J..

[36] Karabicici M, Alptekin S, Firtina Karagonlar Z, Erdal E (2021). Doxorubicin-induced senescence promotes stemness and tumorigenicity in EpCAM-/CD133- nonstem cell population in hepatocellular carcinoma cell line, HuH-7. Mol. Oncol..

[37] Gil A, Plaza-Diaz J, Mesa MD (2018). Vitamin D: Classic and Novel Actions. Ann. Nutr. Metab..

[38] Picca, A., Calvani, R., Coelho-Junior, H. J. & Marzetti, E. Cell Death and Inflammation: The Role of Mitochondria in Health and Disease. *Cells* **10**, 10.3390/cells10030537 (2021). 10.3390/cells10030537 PMC7998762 33802550

[39] Luo, Y., Ma, J. & Lu, W. The Significance of Mitochondrial Dysfunction in Cancer. *Int. J. Mol. Sci.* **21**, 10.3390/ijms21165598 (2020). 10.3390/ijms21165598 PMC7460667 32764295

[40] Baseler WA (2011). Proteomic alterations of distinct mitochondrial subpopulations in the type 1 diabetic heart: contribution of protein import dysfunction. Am. J. Physiol. Regul Integr. Comp. Physiol..

[41] Thapa D (2015). Transgenic overexpression of mitofilin attenuates diabetes mellitus-associated cardiac and mitochondria dysfunction. J. Mol. Cell Cardiol..

[42] Guarani, V. et al. QIL1 mutation causes MICOS disassembly and early onset fatal mitochondrial encephalopathy with liver disease. *Elife* **5**, 10.7554/eLife.17163 (2016). 10.7554/eLife.17163 PMC5021520 27623147

[43] Lamant M (2006). ApoO, a novel apolipoprotein, is an original glycoprotein up-regulated by diabetes in human heart. J. Biol. Chem..

[44] Lee HJ (2011). Downregulation of mitochondrial lon protease impairs mitochondrial function and causes hepatic insulin resistance in human liver SK-HEP-1 cells. Diabetologia.

[45] Sanders FW, Griffin JL (2016). De novo lipogenesis in the liver in health and disease: more than just a shunting yard for glucose. Biol. Rev. Camb. Philos. Soc..

[46] Rada P, Gonzalez-Rodriguez A, Garcia-Monzon C, Valverde AM (2020). Understanding lipotoxicity in NAFLD pathogenesis: is CD36 a key driver. Cell Death Dis..

[47] Chen Y (2019). Mitochondrial Metabolic Reprogramming by CD36 Signaling Drives Macrophage Inflammatory Responses. Circ. Res..

[48] Tang J (2020). Sam50-Mic19-Mic60 axis determines mitochondrial cristae architecture by mediating mitochondrial outer and inner membrane contact. Cell Death Differ.

[49] Chen L (2022). Loss of Sam50 in hepatocytes induces cardiolipin-dependent mitochondrial membrane remodeling to trigger mtDNA release and liver injury. Hepatology.

[50] Li Z (2021). The role of SAMM50 in non-alcoholic fatty liver disease: from genetics to mechanisms. FEBS Open Bio..

[51] Roth CL (2012). Vitamin D deficiency in obese rats exacerbates nonalcoholic fatty liver disease and increases hepatic resistin and Toll-like receptor activation. Hepatology.

[52] Lee YJ, Kim GH, Park SI, Lim JH (2021). Vitamin D Rescues Pancreatic beta Cell Dysfunction due to Iron Overload via Elevation of the Vitamin D Receptor and Maintenance of Ca(2+) Homeostasis. Mol. Nutr. Food Res..

[53] Pines A (2014). Vitamin D and health issues-questioned benefits. Climacteric.

