# Supplementary material for: Vitamin D ameliorates age-induced nonalcoholic fatty liver disease by increasing the mitochondrial contact site and cristae organizing system (MICOS) 60 level
Source: Exp Mol Med. 2024 Jan 4;56(1):142–55. doi: 10.1038/s12276-023-01125-7 (PMC10834941; doi:10.1038/s12276-023-01125-7)
Supplement: Supplementary file 1 — Supplementary Information [file 12276_2023_1125_MOESM1_ESM.pdf]

Supplementary Information

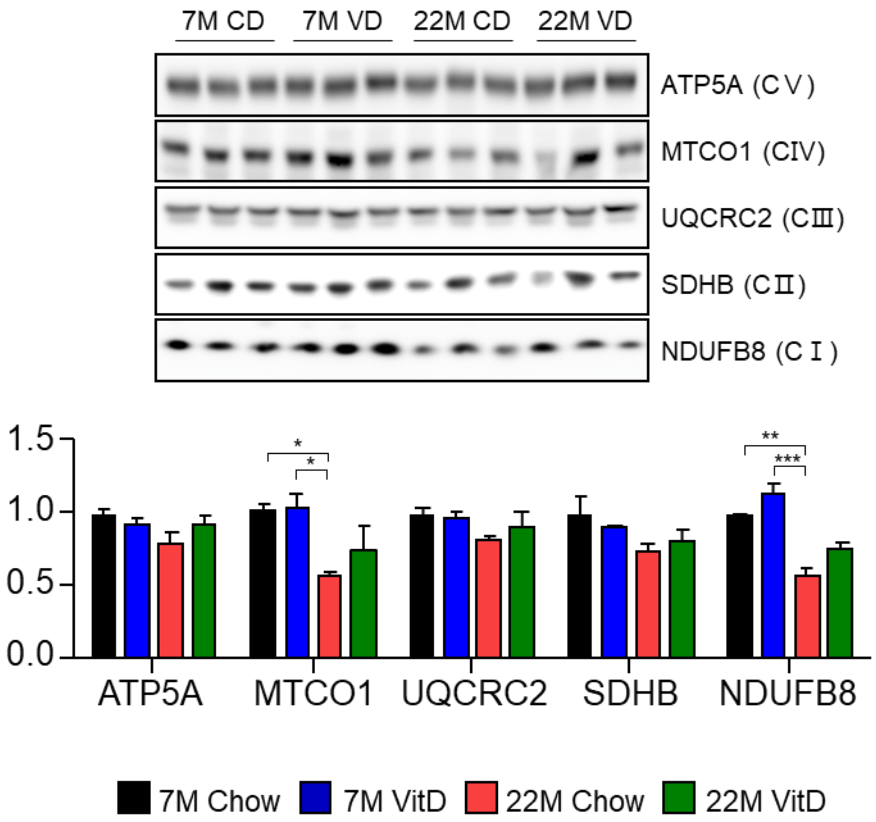

Supplementary Fig. 1. Altered protein levels of OXPHOS in aged liver. OXPHOS protein levels in liver from young and old mice fed the chow and Vitamin D_3-supplemented diet (n=3/group).

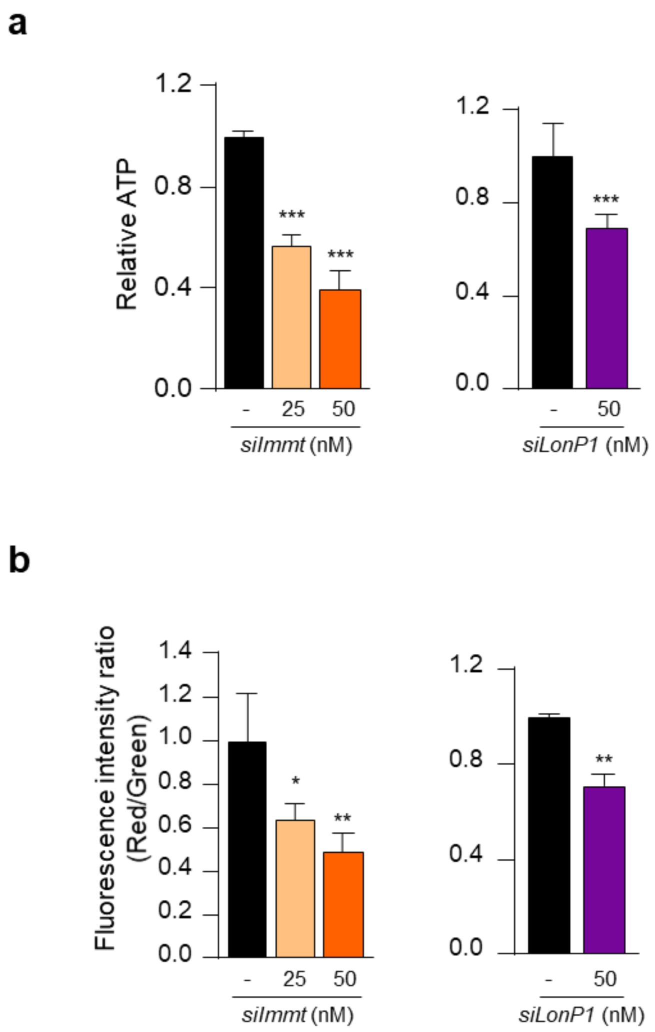

Supplementary Fig. 2. Immt or LonP1 deletion causes mitochondrial dysfunction in HepG2 cells. The intracellular ATP level (a) and mitochondrial membrane potential (b) were measured using a cellular ATP assay kit and JC-1 assay kit, respectively, according to the manufacturer's instructions.

Supplementary Table 1. The Primers used in this study

Primer Name	Sequence (5' to 3')	
mβ-actin	Sense	CACAGCTTCTTTGCAGCTCCT
	Anti-Sense	GTCATCCATGGCGAACTGG
mImmt (MIC60)	Sense	AGGGAGACACTCCAGCTTCA
	Anti-Sense	GACGAGCTGCAACTTCTTCG
mAPOOL (MIC27)	Sense	GCGGCCTTTAGGATGGGAAA
	Anti-Sense	GATCCGGTCTCACTAGCTGC
mCHCHD3 (MIC19)	Sense	TGAAGACAAAGCTCGGCAG
	Anti-Sense	GCTTGAACTTGGCTTCCACC
mMINOS1 (MIC10)	Sense	GAACATCCCAGCGGGAGAAA
	Anti-Sense	ACTGATGGCACTGTCACAGGA
mL-CPT1α	Sense	CTCAGTGGGAGCGACTCTTCA
	Anti-Sense	GGCCTCTGTGGTACACGACAA
mPPARα	Sense	TGCAAACTTGGACTTGAACG
	Anti-Sense	GATCAGCATCCCGTCTTTGT
mFAS	Sense	AAGCCGTTGGGAGTGAAAGT
	Anti-Sense	CAATCTGGATGGCAGTGAGG
mPPARγ	Sense	ACAGGACTGTGTGACAGACA
	Anti-Sense	TATGGGTGAAACTCTGGGAG
mCIDEA	Sense	CCAGAGTCACCTTCGACCTATACA
	Anti-Sense	CATCGTGGCTTTGACATTGAGA
mFSP27	Sense	TCCAGGACATCTTGAAACTT
	Anti-Sense	GGCTTGCAAGTATTCTTCTGT
mGPAT	Sense	TTCCGCCTCTGGGCATT
	Anti-Sense	AGAATCGGCCCACAATCCA
mDGAT	Sense	TTCCGCCTCTGGGCATT
	Anti-Sense	AGAATCGGCCCACAATCCA
mMGAT1	Sense	CTGGTTCTGTTTCCCGTTGT
	Anti-Sense	TGGGTCAAGGCCATCTTAAC
mMGAT2	Sense	GAGCAAAGCCCGTGTGTAGA
	Anti-Sense	AAGGTCTGTAACCTGCGCTC
mCD36	Sense	TGCACCACATATCTACCAAA
	Anti-Sense	TTGTAACCCCACAAGAGTTC
mVLDLR	Sense	GCCATATGAGAACATGCCGC
	Anti-Sense	AGGACACGGGGATACACTGA
mLDLR	Sense	TGACTCAGACGAACAAGGCT
	Anti-Sense	ATCTAGGCAATCTCGGTCTC
mVDR	Sense	GAATGTGCCTCGGATCTGTGG
	Anti-Sense	GGTCATAGCGTTGAAGTGGAA
mCacna1e	Sense	CTGTGCCACCAAAGCCTCGT
	Anti-Sense	CAGGAGGTGGAGAGATGCCG
mItpr1	Sense	TGAAGGGGAACAGAACGAGC
	Anti-Sense	AGGCCGATTCTTTGTTTCTGC
mCalb1	Sense	CCATGCTTAGGCCAGTCAGT
	Anti-Sense	CTTCCGCCAGATAGAAGCAG
mCyp2R1	Sense	GAGGCATATCAACTGTCGTTCT
	Anti-Sense	TGGAATTGAGTAAGCCTCCCA
mCyp27a1	Sense	TCTTCATCGCACAAGGAGAG
	Anti-Sense	ATAACCTCGTTTAAGGCATCC
mCyp2c37	Sense	ATACTCTATATTTGGGCAGG
	Anti-Sense	GTTCCTCCACAAGGCAAC
mCyp3a11	Sense	CTGGGCCCAAACCTCTGCCA
	Anti-Sense	TGTGACAGCAAGGAGAGGCGT
mCyp3a13	Sense	TACCCCAGTATTTGATGCAC

	Anti-Sense	AGATAACTGACTGAGCCACA
hGAPDH	Sense	GTCTCCTCTGACTTCAACAGCG
	Anti-Sense	ACCACCCTGTTGCTGTAGCCAA
hIMMT1	Sense	CACCTGCACTTTCAGAAGCA
	Anti-Sense	CTTGCAGTTTGCCTCAGAGC
hLonP1	Sense	CGGGAAGATCATCCAGTGTT
	Anti-Sense	ACGTCCAGGTAGTGGTCCAG
hPPARα	Sense	GGATGTCACACAACGCGATTC
	Anti-Sense	GGTGGATTGTTACTGGCCTTTC
hL-CPT1a	Sense	GCAGAGGTTCAAGCTGTTCAA
	Anti-Sense	CATGGCTCAGACAGTACCTCCT
hCD36	Sense	CAGGTCAACCTATTGGTCAAGCC
	Anti-Sense	GCCTTCTCATCACCAATGGTCC
hVLDLR	Sense	CAAGGATGGCAGTGATGAGGTC
	Anti-Sense	CTCGGATACCATTACACTGCCTG
hLDLR	Sense	GAATCTACTGGTCTGACCTGTCC
	Anti-Sense	GGTCCAGTAGATGTTGCTGTGG
hFAS	Sense	GGACCCAGAATACCAAGTGCAG
	Anti-Sense	GTTGCTGGTGAGTGTGCATTCC
hPPARγ	Sense	GCAATCAAAGTGGAGCCTGC
	Anti-Sense	TCTCCGGAAGAAACCCTTGC
hCIDEA	Sense	CGGGACTATGCAGGAGCC
	Anti-Sense	GAGGGCATCCAGAGTCTTGC
hFSP27	Sense	AAGCGTGAGGAAGGGCATCATG
	Anti-Sense	CAGTTGTGCCATCTTCCTCCAG
hSREBP1	Sense	ACTTCTGGAGGCATCGCAAGCA
	Anti-Sense	AGGTTCCAGAGGAGGCTACAAG
hSCD1	Sense	CCTGGTTTCACTTGGAGCTGTG
	Anti-Sense	TGTGGTGAAGTTGATGTGCCAGC
hDGAT2	Sense	GCTACAGGTCATCTCAGTGCTC
	Anti-Sense	GTGAAGTAGAGCACAGCGATGAG
hMGAT1	Sense	CCTATGACCGAGATTTCCTCGC
	Anti-Sense	TGAAGCTGTCCCTGCCCGTATA
hMGAT2	Sense	CCAGGGAATTGACAACGTCCTC
	Anti-Sense	AGGGTCACTACCTGGAAACTCG
hCDKN1A	Sense	AGGTGGACCTGGAGACTCTCAG
	Anti-Sense	TCCTCTTGGAGAAGATCAGCCG
hCDKN2A	Sense	CTCGTGCTGATGCTACTGAGGA
	Anti-Sense	GGTCGGCGCAGTTGGGCTCC
hp53	Sense	CCTCAGCATCTTATCCGAGTGG
	Anti-Sense	TGGATGGTGGTACAGTCAGAGC
hIGFBP7	Sense	GCCATCACCCAGGTCAGCAAG
	Anti-Sense	GGATTCCGATGACCTCACAGCT
hIL1A	Sense	TGTATGTGACTGCCCAAGATGAAG
	Anti-Sense	AGAGGAGGTTGGTCTCACTACC
hIL6	Sense	AGACAGCCACTCACCTCTTCAG
	Anti-Sense	TTCTGCCAGTGCCTCTTTGCTG
R1 (-3986~-3203)	Sense	ATTCAATGTCATCTGCCGGGT
	Anti-Sense	ATGACTCCCGTTGCCAATTC
R2 (-3157~-2323)	Sense	AAGGCTTGAGTGCAGTGGTG
	Anti-Sense	GCAGACTGAGGTGGGAGGAT
R3 (-2312~-1724)	Sense	TCAGTCTGCCAAAATGCCGGG
	Anti-Sense	GCTTCAGTCATGTGGTGCCCGT
R4 (-1845~-1159)	Sense	AGGCGCAAGTTGTGGCTGTG
	Anti-Sense	ACGCCATTGCACTCCAGCCT
R5 (-1179~-550)	Sense	AGGCTGGAGTGCAATGGCGT
	Anti-Sense	ACACTGTGGGGACCCTTGTGCT

R6 (-574~115)	Sense	TTAGCACAAGGGTCCCCACA
	Anti-Sense	GCGGTCACACCCGATAACTG
