# Supplementary figures and images for: Vitamin D ameliorates age-induced nonalcoholic fatty liver disease by increasing the mitochondrial contact site and cristae organizing system (MICOS) 60 level
Source: Exp Mol Med. 2024 Jan 4;56(1):142–55. doi: 10.1038/s12276-023-01125-7 (PMC10834941; doi:10.1038/s12276-023-01125-7)

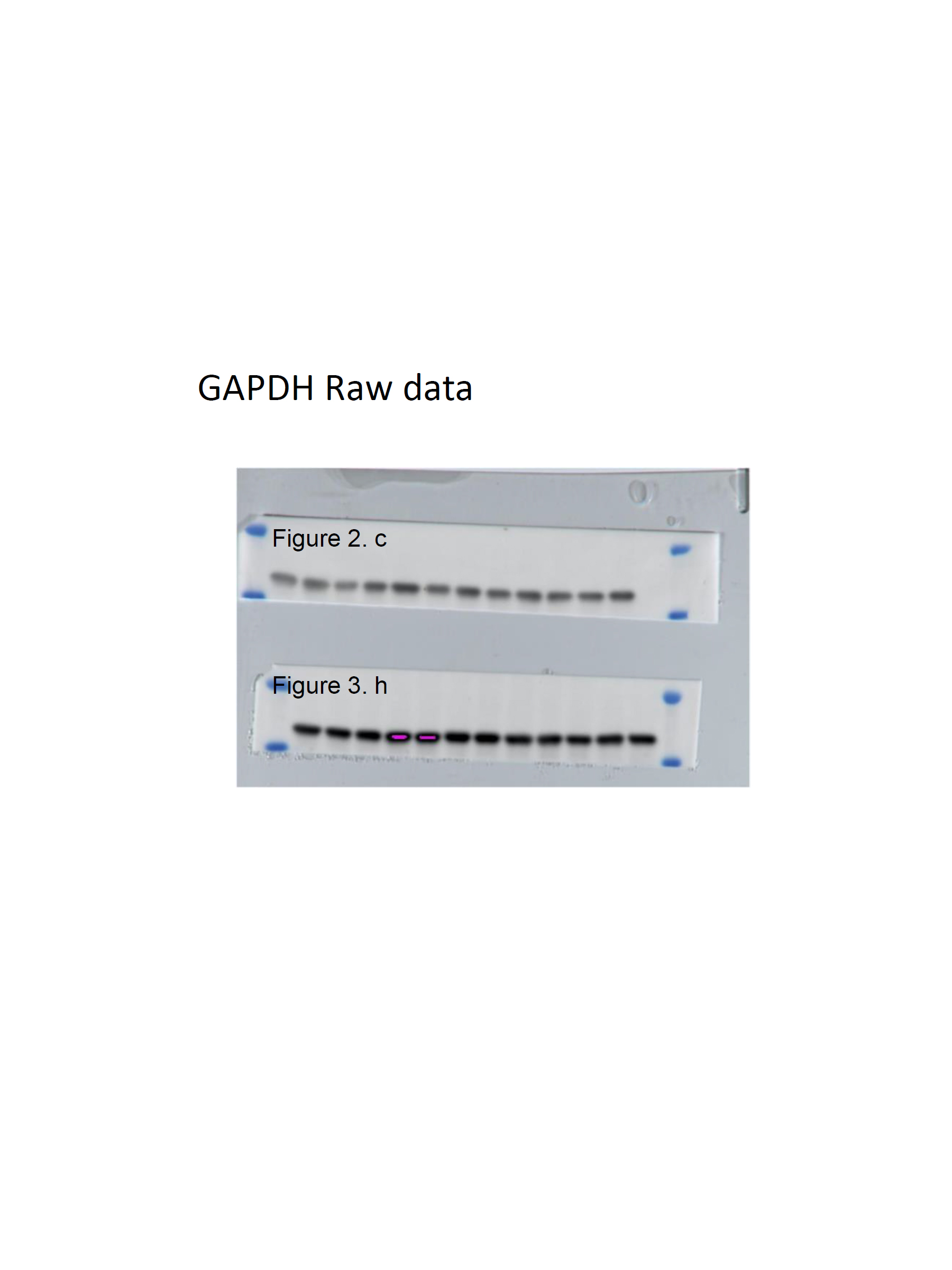

Supplement: Supplementary file 2 — Supplementary Figure for reviewer only [file 12276_2023_1125_MOESM2_ESM.jpg]
